# Molecular identification and phylogenetic analysis of *Cryptosporidium*, *Hepatozoon* and *Spirometra* in snakes from central China

**DOI:** 10.1016/j.ijppaw.2019.10.001

**Published:** 2019-10-09

**Authors:** Xiao Xiao, Rui Qi, Hui-Ju Han, Jian-Wei Liu, Xiang-Rong Qin, Li-Zhu Fang, Chuan-Min Zhou, Xiao-Qing Gong, Si-Cong Lei, Xue-Jie Yu

**Affiliations:** a State Key Laboratory of Virology, School of Health Sciences, Wuhan University, Wuhan, PR China; b Lab Animal Research Center, Hubei University of Chinese Medicine, Wuhan, China

**Keywords:** Zoonotic, *Cryptosporidium*, *Hepatozoon*, *Spirometra*, Snake

## Abstract

Snakes are popular as food and traditional medicine in China. However, information about parasitic and bacterial infections in snakes from China is scarce. We investigated the prevalence of selected zoonotic agents including *Cryptosporidium*, *Hepatozoon* and *Spirometra*, in snakes in central China from June to October in 2018 by PCR amplification using parasite-specific primers. PCR amplification and DNA sequencing showed that 10.1% (15/149) of snakes were positive for *Cryptosporidium* spp., while 2.7% (4/149) were positive for *Hepatozoon*. Additionally, we found 36.9% (55/149) of snakes were infected with *Spirometra erinaceieuropaei*. The spargana burden per infected snake ranged from 1 to 26. BLAST and phylogenetic analysis of small subunit ribosomal RNA (SSU rRNA) gene and 60-kDa glycoprotein (*gp60*) gene showed that the parasites belonged to *Cryptosporidium parvum* genotype IIdA15G1, *C. baileyi*, *C. serpentis* and a *Hepatozoon* species. We conclude that intensively farmed snakes excrete *C. parvum* and *C. baileyi* oocysts due to ingestion of infected feeder animals, and that wild snakes in central China were commonly infected with *S. erinaceieuropaei*, suggesting that eating improperly cooked snakes could be risky to human health.

## Highlights

• *Cryptosporidium parvum* and *C. baileyi* were reported in farmed snakes in China for the first time.
• *C. serpentis* was detected for the first time in *Ptyas dhumnades* and *Gloydius brevicaudus.*
• A tentative new *Hapatozoon* species was first time recorded in wild *Lycodon rufozonatus* and *G. brevicaudus.*
• *Spirometra erinaceieuropaei* infection was found common in wild snakes from central China.

## Graphical abstract

**Figure:**
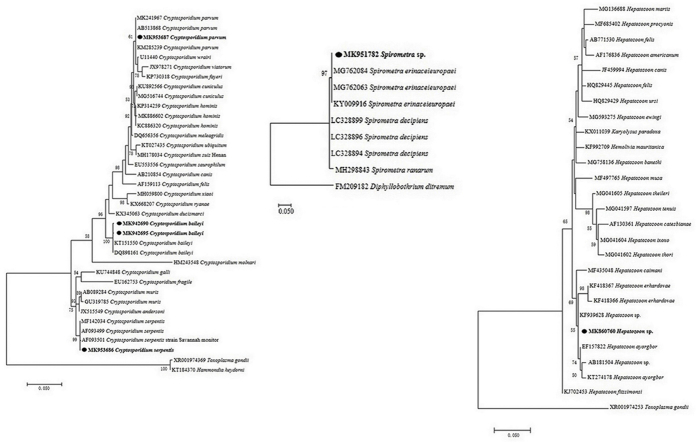

## 1 Introduction

Snakes have long been considered a delicacy and traditional medicine in China and other Asian countries (Zhou and Jiang, 2005). Previous surveys showed that snake farming in China and southeast Asia has greatly increased over the last twenty years, and the total quantity of snakes traded in China each year is estimated to be 7000–9000 tons (Aust et al., 2017; Zhou and Jiang, 2004). More than 3600 tons of snakes were consumed annually in a single Chinese city, Guangzhou (Wang et al., 2011, 2014a). Snakes have been confirmed worldwide as hosts of parasites and several neglected foodborne zoonoses (Tang et al., 2017; Tomé et al., 2012; Yimming et al., 2016). Snakes represent a very diverse group of vertebrates in China, with more than 200 species found (Zhou and Jiang, 2005). However, little information in China is available about their parasites in comparison with mammals and birds.

Cryptosporidiosis is a severe diarrheal disease caused by *Cryptosporidium* (Xiao et al., 2004). The protozoans live in the intestine of various vertebrate hosts including humans and snakes. *Cryptosporidium* infections have been characterized by fever, lethargy, anorexia, hemorrhagic watery diarrhea and even death in humans and mammals (Navin and Juranek, 1984; O'Donoghue, 1995). Infective oocysts are passed in the stool of an infected person or animal. *Cryptosporidium serpentis* and *C. varanii* (syn. *C. saurophilum*) have been confirmed in snakes. *Cryptosporidium varanii* infection is subclinical in snakes, while *C. serpentis* infection presents with anorexia, weight loss, food regurgitation, and mid-body swelling, and may bring devastating economic losses to the snake farming industry (Paiva et al., 2013; Yimming et al., 2016).

*Hepatozoon* is a common intracellular protozoan parasite found in reptiles, birds, and mammals. Several tick-borne *Hepatozoon* species are the causal agents of Hepatozoonosis in dogs (Baneth et al., 2003; Harvey, 2012). More than 120 *Hepatozoon* species have been described in more than 200 snake species (Han et al., 2015). The protozoans exhibit heteroxenous life cycles involving tissue merogony and gametogony within the vertebrate host and a sexual cycle and sporogony within the invertebrate host. *Hepatozoon* transmission in vertebrates occurs by intrauterine transmission, the ingestion of infected ticks or by the predation of other infected vertebrates (David et al., 2017; Smith, 1996). The pathogenic potential of *Hepatozoon* spp. in snakes is still controversial (Brown et al., 2006; Madsen et al., 2005). Previous studies have suggested that different infection levels of *Hepatozoon* may have widely differing effects on snakes, ranging from trivial consequences for host fitness to severe effects on growth rate and reproductive output (O'Dwyer et al., 2013; Tomé et al., 2012). Studies of these parasites are, therefore, necessary not only to improve our understanding of this component of biodiversity but also to assess any potential risk to host populations.

Sparganosis is a neglected zoonosis caused by the plerocercoid larvae (spargana) of the genus *Spirometra*. Species of *Spirometra* are distributed worldwide but human infections have mostly been reported in Asia (Liu et al., 2015; Tang et al., 2017). Spargana live in second intermediate hosts, mainly including amphibians and reptiles. Human beings are commonly infected through consumption of raw or half cooked frogs and snake meat, or by using the raw flesh of these animals as poultices in traditional medicine (Liu et al., 2015). The consumption of snakes has been increasing in the last two decades, and a recent study reported a high prevalence of *Spirometra* in both captive and wild snake species from southern China (Wang et al., 2011, 2014a).

Despite the medical and veterinary importance of the above parasites, the prevalence and taxonomy of these groups in snakes are still poorly understood. Both *Hepatozoon* and *Cryptosporidium* infections in snakes are difficult to diagnose, especially in those with asymptomatic infections. Differentiation among species of *Hepatozoon* gamonts present within blood cells, or species of *Cryptosporidium* oocysts in the intestines of snakes is extremely difficult because of high morphological similarity within a genus. (Han et al., 2015; Yimming et al., 2016). Even with good training, species identification by morphology is likely extremely difficult, and often not possible. Likewise, the morphological identification of spargana is also difficult because different species lack distinguishing characteristics (Almeida et al., 2016).

Molecular epidemiology has played an important role over the decades in parasite studies and helped ensure accurate species identification pertaining to phylogenetics, genetic variation and evolution. The objective of the present study was to determine the prevalence, taxonomy and phylogenetic relationship of *Cryptosporidium*, *Hepatozoon* and *Spirometra* in farmed and wild-caught snakes in Hubei Province, central China, based on the polymerase chain reaction (PCR) method. The species of identified parasites was ascertained by phylogenetic analysis of targeted gene sequences of the examined samples.

## 2 Material and methods

Between June and October of 2018, 149 snakes of 13 species were collected from two seafood markets in Wuhan City from Hubei Province, China. According to the transportation records of the dealers, 99 specimens of 8 species were wild-collected in Hubei Province and 50 specimens of 5 species were captive bred and raised in farms from unknown localities in Hunan Province. We followed the Lillywhite guidelines in preparing the snakes (Lillywhite et al., 2016). The head of the snake was immersed in liquid nitrogen with a snake tong, which resulted in rapid freezing and death. Destruction of brain tissue (pithing) was immediately followed. For specimens longer than 1 m, cardiocentesis was immediately performed for blood collection. For smaller snake specimens, the tails were removed, and approximately 1–2 mL of blood samples were immediately collected. Large intestinal contents or fecal samples from each specimen were collected and stored at 4 °C after being mixed with an equal volume of 5% potassium dichromate. *Spirometra* infection was thoroughly examined following the guideline of Wang (Wang et al., 2011). Sparganas from the same snake species were grouped together and the number was recorded.

### 2.1 Microscopy screening and concentration of *Cryptosporidium*

Fecal or large intestinal content samples were mixed with phosphate-buffered saline (PBS) to make homogeneous suspensions. The suspensions were sequentially sieved and finally purified through 63-μm porosity mesh stainless steel screens. After concentration using Sheather's discontinuous sucrose gradient centrifugation technique as previously described (Arrowood, 2020), all supernatants were examined by optical microscopy under 250× magnification for the screening of *Cryptosporidium* oocysts based on the morphological standard previously reported (Arrowood and Sterling, 1987).

### 2.2 DNA extraction

The supernatants containing *Cryptosporidium* oocysts were repeatedly frozen and thawed in liquid nitrogen and a 65 °C water bath, respectively, before centrifugation (Wang et al., 2014b). The total DNA was extracted using the QIAamp® DNA Stool Mini Kit (Qiagen, Germany) according to the manufacturer's instructions. For blood samples, genomic DNA was extracted using a Qiagen DNeasy Blood & Tissue Kits (Qiagen, Germany) according to the manufacturer's instructions. Total genomic DNA of sparganas was extracted from representative sparganas isolated from different infected snake species using a DNeasy Tissue Kit (Qiagen, Germany) according to the manufacturer's instructions. All purified DNA samples were stored at −20 °C for further molecular analysis.

### 2.3 PCR amplification and sequencing

Nested PCR was performed to identify *Hepatozoon* and *Cryptosporidium* species based on the 18S small subunit rRNA (SSU rRNA) gene sequence (Aydin et al., 2015; Sumrandee et al., 2015; Xiao et al., 2004). DNA from *Cryptosporidium* SSU rRNA-positive samples was subjected to further PCR-based analysis targeting the 60-kDa glycoprotein (*gp60*) gene (Alves et al., 2003). The highly conserved *cox1* gene was targeted for identification of *Spirometra* species using conventional PCR (Lee et al., 2007). The primer sequences, product lengths, and the annealing temperatures are listed in Table 1. The previously isolated DNA of *Spirometra erinaceieuropaei* and *Cryptosporidium parvum* were used as positive controls, while reagent-grade water served as a negative control in each run. All steps were performed in separate rooms to avoid contamination. The secondary PCR products were examined by electrophoresis using a 1.2% agarose gel and staining with ethidium bromide, and observed under UV light. PCR products with expected sizes were excised from gels and extracted using a Gel Extraction Kit (Promega, Madison, WI, USA). The PCR products were cloned into the pMD 19-T vectors (TaKaRa, Shiga, Japan) and recombinant clones were sequenced bidirectionally with Sanger sequencing.

**Table 1:** PCR primers used in identification of *Cryptosporidium*, *Hepatozoon* and *Spirometra*.

Organisms	PCR method	Primer	Primer sequences (5'→3′)	Target gene	Annealing temp (°C)	Amplicon size (bp)	References
*Spirometra*	PCR	JB3	TTTTTTGGGCATCCTGAGGTTTAT	*cox1*	55	~446	Lee et al. (2007)
		JB4.5	TAAAGAAAGAACATAATGAAAATG				
*Cryptosporidium*	Nested PCR	SSU-F2	TTCTAGAGCTAATACATGCG	SSU rRNA	55	~1325	Xiao et al. (2000)
		SSU-R2	CCCATTTCCTTCGAAACAGGA				
		SSU-F3	GGAAGGGTTGTATTTATTAGATAAAG		55	~820	Xiao et al. (2004)
		SSU-R3	AAGGAGTAAGGAACAACCTCCA				
	Nested PCR	AL3531	ATAGTCTCCGCTGTATTC	*gp60*	55	~1280	Alves et al. (2003)
		AL3535	GGAAGGAACGATGTATCT				
		AL3532	TCCGCTGTATTCTCAGCC		58	~850	
		AL3534	GCAGAGGAACCAGCATC				
*Hepatozoon*	Nested PCR	HepF300	GCTAATACATGAGCAAAATCTCAA	18S rRNA	54		(Aydin et al., 2015; Sumrandee et al., 2015)
		HepR900	CGGAATTAA CCAGACAAAT				
		HepF	ATACATGAGCAAAATCTCAAC		59	~640	
		HepR	CTTATTATTCCATGCTGCAG				

### 2.4 Phylogenetic analysis

Sequences were aligned with ClustalW from MEGA 7.0 and searched using BLAST in the GenBank database (Kumar et al., 2016). The evolutionary models for datasets including representative and downloaded reference sequences were estimated using jModeltest2 (Darriba et al., 2012). Phylogenetic trees were constructed using the Maximum likelihood method in MEGA 7.0, and the robustness of the trees was tested with 1000 bootstrap replications. *Toxoplasma gondii* (XR001974253), *Hammondia heydorni* (KT184370) and *Diphyllobothrium ditremum* (FM209182) were set as out-groups. Genotypes of *Cryptosporidium* were determined after alignment with reference sequences downloaded from GenBank using ClustalX 2.1 (http://www.clustal.org/).

## 3 Results

### 3.1 Genotyping and subtyping *Cryptosporidium*

**Fig. 1:**
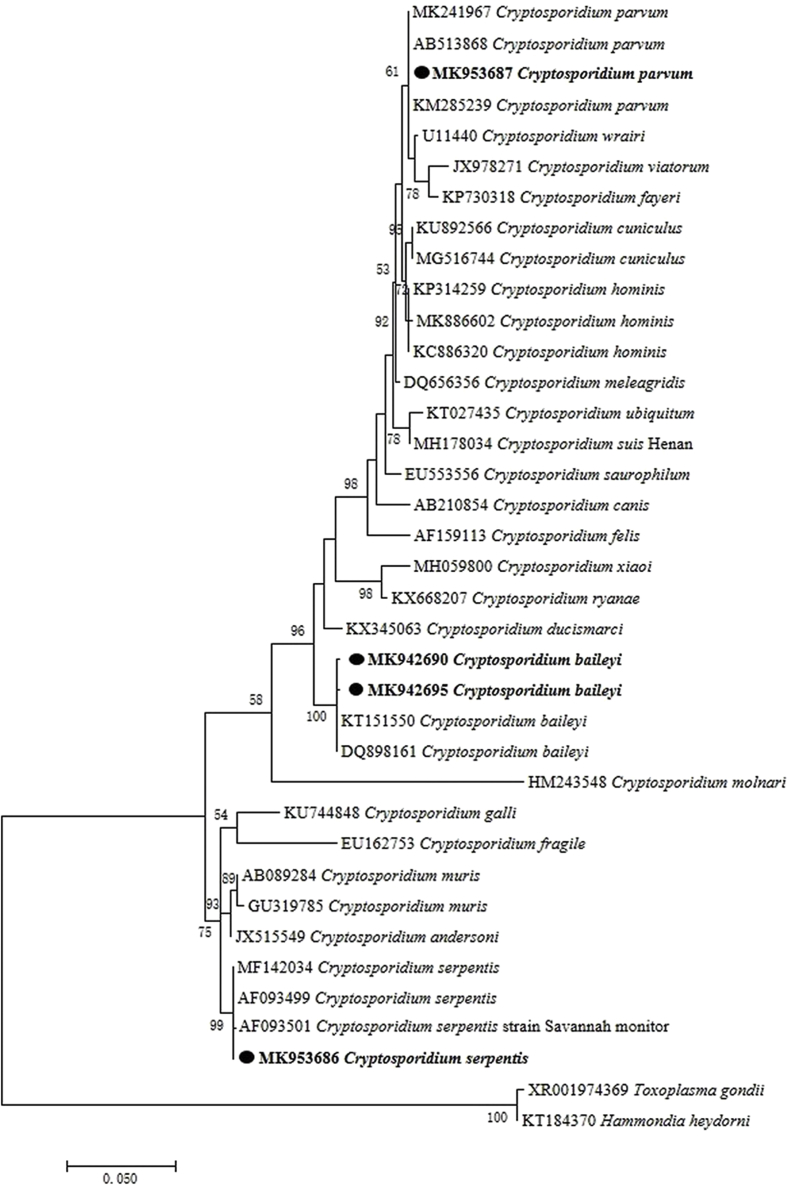
Maximum likelihood phylogenetic tree based on the SSU gene of *Cryptosporidium*. The phylogenetic tree SSU gene (834bp) was constructed by using the Kimura 2-parameter model with MEGA 7.0 and the bootstrap values were calculated with 1000 replicates. Representative sequences of *Cryptosporidium* detected in snakes in this study are in bold print and marked by circles. Scale bar indicates nucleotide substitutions per site.

Among 149 snakes, 15 were positive to *Cryptosporidium* with an infection prevalence of 10.1% (Table 2). BLAST analysis indicated that the sequences from the snakes belonged to three *Cryptosporidium* species. Ten sequences were 99.88–100% identical to each other and were identified as *C. baileyi* (6.7%, 10/149), three were 100% identical to *C. parvum* (2%, 3/149) and two were 100% identical to *C. serpentis* (1.3%, 2/149). Phylogenetic analysis using the four representative sequences also showed *Cryptosporidium* identifies in the present study clustered with *C. baileyi*, *C. parvum* and *C. serpentis* respectively (Fig. 1). The three *C. parvum* sequences had 100% homology to previous isolates derived from dairy cattle (MF671870) in China. Of the ten *C. baileyi* sequences, seven were 99.88% homologous to previous isolates from quails (DQ89816) in China, and three were 100% homologous to previous isolates from chickens (JX548296) in Zhejiang Province and ducks (AY954882) in Henan Province, China. The two *C. serpentis* sequences were 100% homologous to previous isolates derived from the oriental rat snake *Ptyas mucosus* (KJ651433). The *C. parvum* samples were further subtyped with *gp60* gene sequence. Two positive samples were 100% homologous with previous sequences of subtype IIdA15G1 from sheep (MH794167) in Xinjiang, China.

**Table 2:** Infection of *Cryptosporidium*, *Hepatozoon* and *Spirometra* in snake species in central China.

Snake species	Number	Main prey or farm feeding items	Number of Positive Sample				
			Spirometra	Cryptosporidium			Hepatozoon
				C. parvum	C. baileyi	C. serpentis	
**Captive raised**							
*Elaphe carinata*	12	Farm supplied quick-frozen defeathered young chicks of duck, quail, layer fowl and broiler fowl, eggs, etc.	0	0	3	0	0
*Naja atra*	13		0	0	1	0	0
*Naja kaouthia*	7		0	0	2	0	0
*Ptyas mucosa*	12		0	2	4	0	0
*Deinagkistrodon acutus*	6	Rodents, frogs, young chicks of duck, quail, layer fowl and broiler fowl, etc.	2	1	0	0	0
**Wild caught**							
*Bungarus multicinctus*	8	Frogs, rodents, snakes, pond loach, swamp eel, etc.	3	0	0	0	0
*Lycodon rufozonatus*	12	Frogs, snakes, rodents, birds, fish,etc.	3	0	0	0	3
*Orthriophis taeniurus*	19	Rodents, shrews, birds, etc.	11	0	0	0	0
*Ptyas dhumnades*	25	Frogs, rodents, birds, etc.	23	0	0	1	0
*Oocatochus rufodorsatus*	9	Frogs, fish, etc.	2	0	0	0	0
*Sinonatrix annularis*	12		1	0	0	0	0
*Euprepiophis mandarinus*	2	Small rodents, shrews, etc.	0	0	0	0	0
*Gloydius brevicaudus*	12	Frogs, rodents, shrews, birds, etc.	10	0	0	1	1
**Total Number**	**149**		**55**	**3**	**10**	**2**	**4**

### 3.2 Occurrence of *Hepatozoon*

**Fig. 2:**
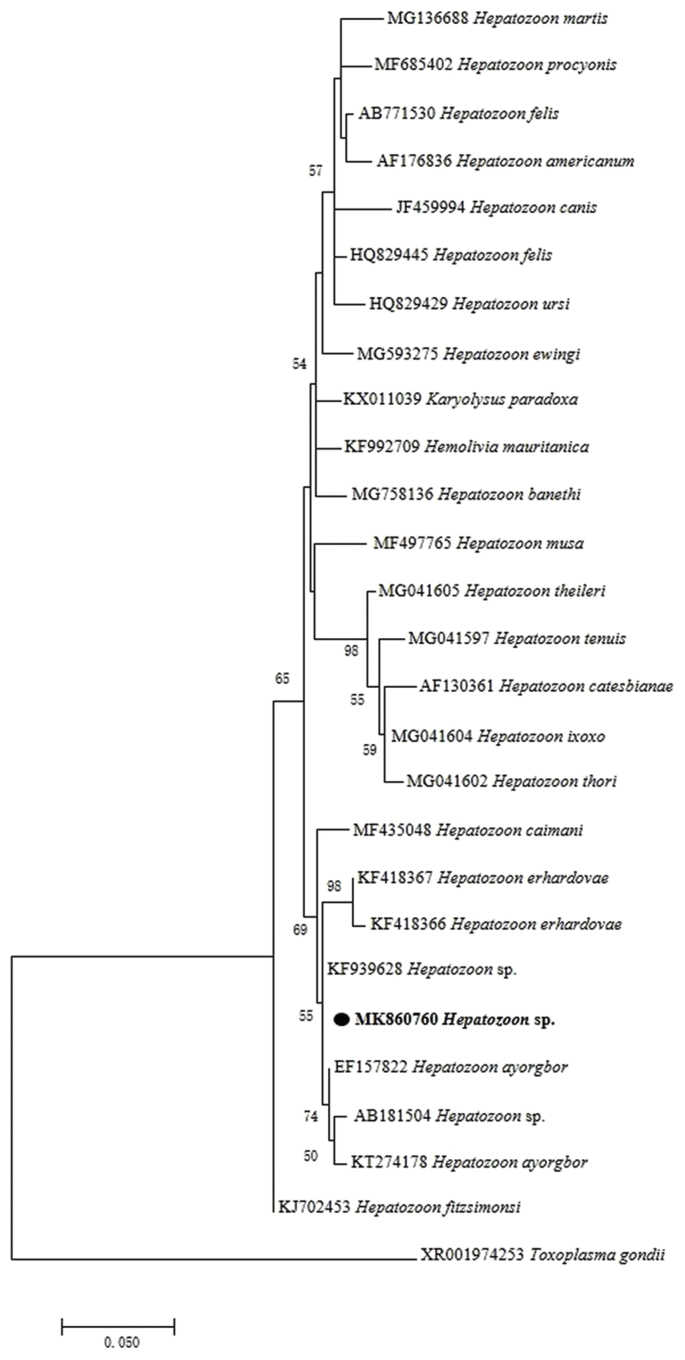
Maximum likelihood phylogenetic tree based on the 18S rRNA gene of *Hepatozoon*. The phylogenetic tree was constructed with the 18S rRNA gene sequences (670bp) by using the General time reversible model with MEGA 7.0; the bootstrap values were calculated with 1000 replicates. Representative sequences of *Hepatozoon* detected in this study are in bold print and marked by circles. Scale bar indicates nucleotide substitutions per site.

*Hepatozoon* DNA was detected in blood samples of *L. rufozonatus* and *G. brevicaudus* by nested PCR and sequencing. Four 18S rRNA DNA sequences obtained in this study were 100% identical to each other, and were 99.4% identical to corresponding regions of *H. ayorgbor* isolated from the house snake (*Lamprophis fuliginosus*, EF157822) in Ghana, and 98.51% identical to *H. ayorgbor* isolated from the greater bandicoot rat (*Bandicota indica*, AB181504) in Chiang Mai, Thailand (Fig. 2). Sequences analysis showed a 529bp fragment of the representative sequence had 100% similarity with partial SSU sequence from the king rat snake (KF939627) in Shanghai.

### 3.3 Infection of *Spirometra*

**Fig. 3:**
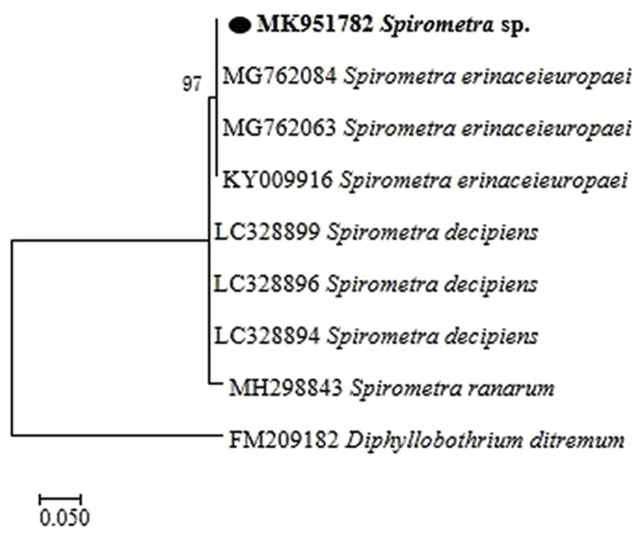
Maximum likelihood phylogenetic tree of *Spirometra* based on the *cox1* gene. The tree was constructed with the *cox1* sequences (444bp) by using the Kimura 2-parameter model with MEGA 7.0; we calculated bootstrap values with 1000 replicates. The representative sequence of *Spirometra* spagarnas isolated from snakes in this study are in bold print and marked by circles. Scale bar indicates nucleotide substitutions per site.

55 snakes of 8 species were found infected with spargana, with the highest infection rate being recorded in *P. dhumnades* (92%, 23/25), followed by *G. brevicaudus* (83.3%, 10/12). A total of 339 sparganas were collected. The spargana burden per snake ranged from 1 to 26. A total of 40 representative spargana samples were randomly chosen from 8 infected snake species group for DNA extraction. Sequence analysis of mitochondrial *cox1* gene indicated all 40 obtained sequences share 100% similarity with *S. erinaceieuropaei* (MG762084) recovered from snakes in Hunan Province, China. Phylogeny analysis also showed the spargana recovered in the present study clustered with *S. erinaceieuropaei* (Fig. 3).

## 4 Discussion

There is a long history of exploitation of snakes in Asia in traditional medicine and as food. Snake farming is now extensive in China since the dietary habit has spread all over the country and was boosted by rapid economic growth (Aust et al., 2017). On three farms alone in Guangdong Province there were 40,000 captive-bred oriental rat snakes in a single year and captive bred snakes were occasionally released to reinforce the wild population (Jiang et al., 2013). However, information related to neglected zoonotic agents in both captive and wild snakes in China is very limited despite its importance to public health and snake conservation.

In the present study, we reported for the first time *C. baileyi* in snakes and *C. parvum* genotype IIdA15G1 in *D. acutus*. *Cryptosporidium serpentis* was reported for the first time in wild *P. dhumnades* and *G. brevicaudus*. Notably, all *C. baileyi* and *C. parvum* positive individuals were captive-bred and raised. Previous studies and our survey confirmed *E. carinata*, *N. atra*, *N. kaouthia* and *P. mucosa* are the most extensively farmed snake species since they accept dead or defrosted feeder animals, and thus are easy to be intensively farmed (Aust et al., 2017). All five captive snake species in our study were maintained on a diet of farm supplied quick-frozen defeathered young chicks of ducks, quails, and layer fowls along with their eggs. The previous survey indicated a 7.0% prevalence of *C. baileyi* in farmed chickens in Hubei Province, while *C. parvum* has also been reported in rodents and goats from neighboring areas (Chai et al., 2019; Liao et al., 2018; Mi et al., 2014). Experimental infection in laboratory settings confirmed *C. baileyi* and *C. parvum* were noninfectious to snakes (Graczyk and Cranfield, 1998). Thus, our study suggested a predator-prey transmission of *Cryptosporidium* in snakes and the fact that *Cryptosporidium*-infected feeder animals can sustain a source of oocysts that are passively transferred through snakes. Even if the snake is not infected, these oocysts can be detected in their stools. The occurrence of *Hepatozoon* in Hubei region, central China has never been investigated. Only two *Hepatozoon* species have been previously reported in snakes from China, even though a high diversity of *Hepatozoon* spp. have been reported in various snake species throughout the world (Cook et al., 2018; Han et al., 2015; Tomé et al., 2012; Tome et al., 2014). In 1987, Indo-Chinese rat snakes (*Ptyas korros*), radiated rat snakes (*Coelognathus radiata*), freshwater snakes (*Xenochrophis piscator*) and Chinese water snakes (*Enhydris chinensis*) have been reported to be infected with *H. guangdongense*, which has been described based on microscopic evidence. A new species, *H. chinensis* has been isolated from king rat snakes (*E.carinata*) in 2015 (Han et al., 2015). In the present study, the obtained *Hepatozoon* SSU sequence has a 529bp 100% identical overlapping region with a sequence previously obtained from *H. chinensis*. The whole sequence was 98.51–99.4% identical to *H. ayorgbor* isolated from the house snake (*Lamprophis fuliginosus* EF157822) in Ghana, and the greater bandicoot rat (*Bandicota indica* AB181504) in Chiang Mai, Thailand. The sequence also clustered with *H. ayorgbor* and *H. chinensis* in phylogenetic analysis. We also tried to further molecularly identify the *Hepatozoon* detected in this study with primers HEMO1/HEMO2 targeting different part of the 18S rRNA gene. However, no sequence was obtained. Thus the taxonomic position of this *Hepatozoon* species could not be ascertained and needs to be further confirmed based on more morphologic and molecular evidence. Considering that vertical transmission of *Hepatozoon* was confirmed in the Garter Snake (*Thamnophis elegans*) and all *Hepatozoon* infected snakes in our study were asymptomatic (Kauffman et al., 2017), the pathogenic potential and possibility of vertical transmission of present *Hepatozoon* sp. in snakes also needs to be further investigated.

Phylogenetic analysis indicated all the sparganas isolated in our study were *S. erinaceieuropaei*. Our results showed that *P. dhumnades* had the highest infection rate, which is consistent with the survey results of Wang et al. in Guangzhou of Guangdong Province (Wang et al., 2011). Interestingly, none of the *E. carinata*, *N. atra*, *N. kaouthia* and *P. mucosa* were found infected by spargana, while three out of these species have been previously reported with infection rates between 7.1 and 87.5% (Wang et al., 2011). *Deinagkistrodon acutus* is the only farm raised species infected with spargana in our study. This result suggests that the *Spirometra* infection in farmed snakes was closely related to those in prey animal sources. All non-infected snakes in our study were farmed snakes and they were never fed with frogs. *Deinagkistrodon* *acutus* shows strong prey specificities in captivity, and some individuals only prey on frogs in certain life stages. Meanwhile, we found none of *E. mandarinus* examined were infected with spargana but infection in *P. dhumnades* was common (23/25, 92%). *Euprepiophis mandarinus* has never been observed preying on frogs, but *P. dhumnades* often preys on frogs. Sparganum infection rates in wild frogs in central, eastern, southern and southwest China are high (Zhang et al., 2015, 2016). Previous studies also indicated *Spirometra* infection in snakes is closely related to those in prey animal sources especially frogs (Wang et al., 2011). Nevertheless, the result of our study may be due to the genetic susceptibility of *P. dhumnades* to sparganum infection or be biased due to the small sample size.

## 5 Conclusion

To the best of our knowledge, this is the first time *C. parvum* and *C. baileyi* were reported in farmed snakes in China and *C. serpentis* was detected in *P. dhumnades* and *G. brevicaudus*. Our study was also the first time a *Hepatozoon* species was recorded in wild *L. rufozonatus* and *G. brevicaudus*. *Spirometra* *erinaceieuropaei* infection was commonly found in wild snakes from central China. We conclude that intensive snake farming may enhance transmission of *Cryptosporidium* and *Hepatozoon* between snakes and therefore needs to be regulated. Our study provides new data on these zoonotic agents in this region. The diversity and transmission characteristics of these agents and their implications to public health still need to be further investigated.

## Ethical approval

This study was approved by the Ethics Committee of Wuhan University (2018010). Snakes were handled in accordance with good animal practices required by the Animal Ethics Procedures and Guidelines of the People's Republic of China.

## Data availability

The representative SSU rRNA and *cox1* gene sequences obtained in this study were deposited in GenBank under the following accession numbers: MK953687, MK942690, MK942695, MK953686, MK860760, and MK951782.

## Declaration of competing interest

The authors declare that they have no competing interests.
